# Tirzepatide: A Novel, Once-weekly Dual GIP and GLP-1 Receptor Agonist for the Treatment of Type 2 Diabetes

**DOI:** 10.17925/EE.2022.18.1.10

**Published:** 2022-06-16

**Authors:** Shizuka Kaneko

**Affiliations:** Division of Diabetes/Endocrinology/Lifestyle-Related Disease, Takatsuki Red Cross Hospital, Takatsuki, Japan

**Keywords:** Gastric inhibitory polypeptide (GIP), glucagon-like peptide-1 (GLP-1), dual agonist, incretin, type 2 diabetes mellitus, tirzepatide, SURPASS

## Abstract

Gastrointestinal hormones are currently used to treat type 2 diabetes mellitus (T2D). Incretin preparations with gastric inhibitory polypeptide (GIP) activity or glucagon-like peptide-1 (GLP-1) provide new means for controlling blood glucose levels, body weight, and lipid metabolism. GIP, an incretin, has not been used due to lack of promising action against diabetes. However, recent studies have shown that GIP has an important effect on glucagon and insulin secretion under normoglycaemic conditions. Co-existence of GIP with GLP-1 and glucagon signalling leads to a stronger effect than that of GLP-1 stimulation alone. The development of a GIP/GLP-1R unimolecular dual agonist with affinity for both GIP and GLP-1 receptors is under investigation, and the drug is expected to be clinically available in the near future. Tirzepatide, a GIP/GLP-1R unimolecular dual agonist, regulates metabolism via both peripheral organs and the central nervous system. The SURPASS phase III clinical trials conducted for tirzepatide comprise 10 clinical trials, including five global trials and the global SURPASS-CVOT trial, with >13,000 patients with T2D (ClinicalTrials.gov Identifier: NCT04255433). The clinical application of tirzepatide as a therapy for T2D may provide new insights into diabetic conditions and help clarify the role of GIP in its pathogenesis.

Nutrient-stimulated gastric inhibitory polypeptide (GIP) (known as either gastric inhibitory polypeptide or glucose-dependent insulinotropic polypeptide) or glucagon-like peptide (GLP-1), are secreted by K and L cells, respectively, in the upper segment of the small intestine and throughout the intestine.

For the past 10 years, GLP-1 receptor agonists, which are mimetic of incretins, have been used for the treatment of type 2 diabetes mellitus (T2D). GIP as a drug has not been developed due to lack of promising action against diabetes.^1,2^ After the discovery of its amino acid sequence in 1971, GIP was found to promote insulin secretion in a glucose-dependent manner. By cloning the GIP cDNA, the structure of prepro-GIP was successfully revealed.^3^ GIP mRNA expression in the upper segment of the small intestine and localization of its gene in the chromosome was demonstrated.^4^ GIP is shown to be related to high-fat diet-induced obesity.^5^

Functional analysis of GIP reports its potential effect on glucagon and insulin secretion under normoglycaemic conditions.^6^ It also has positive effects on lipid metabolism, bone metabolism and the central nervous system (CNS). Moreover, the effect of GIP, GLP-1 and glucagon signalling together is stronger compared to GLP-1 alone. Succeeding this work, a unimolecular dual agonist of GIP and GLP-1 is under development and will be available in the near future as anticipated. Treatments that regulate metabolism, particularly gastrointestinal hormones, are becoming increasingly common; for example, GIP plus glucagon or a triagonist comprising GIP, GLP-1 and glucagon is also being developed for patients with obesity, diabetes or dyslipidaemia. Herein, we review GIP activation based on *in vivo* studies, focusing on the physiological role of cooperative GIP, and SURPASS, which include 10 clinical studies of tirzepatide–GIP/GLP-1 dual agonist.

Based on the treatment outcomes with GIP/GLP-1 dual agonist, we might explore other combinations, such as a triagonist–GLP/GIP/glucagon or other dual agonists involving glucagon, which are currently under development.

## Gastrointestinal hormones, incretin, GIP and GLP-1

Nutrients in the digestive tract stimulate the secretion of gastrointestinal hormones and activate metabolic function.^1,7,8^ Subsequently, this influences immunity and is involved in communication among organs.

**Figure 1:**
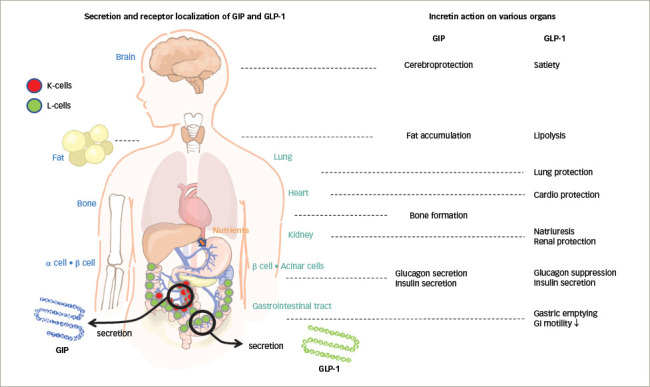
GIP and GLP-1 secretion acting on receptors in various organs^10–14^

Gastrointestinal hormones - especially GIP and GLP-1 - both gut-induced incretins, are secreted in response to nutrient ingestion, which stimulates the pancreatic β-cells to increase insulin secretion in a glucose-dependent manner; thus, exhibiting insulinotropic effects.^7,8^ GIP and GLP-1 are occasionally secreted by the same cell, also referred to as K/L or L/K cells (*Figure 1*).^7–14^ The influence of GIP and GLP-1 on the pancreatic α-cell function is inverse. GIP stimulates the α-cells to promote glucagon secretion, while GLP-1 reduces it.^10,15^ Elevation of glucagon is associated with T2D.^16^ Therefore, suppression of glucagon activity using a GLP-1 receptor (GLP-1R) agonist has been developed for treatment of T2D.^17^ The use of gastrointestinal hormones, particularly GLP-1 and GIP, includes incretin preparations such as a novel GLP-1R agonist or a GIP/GLP-1 dual agonist, which is anticipated to provide new means for controlling T2D and body weight.^18–20^

*GIP (secreted by K-cells in the upper segment of the small intestine) and GLP-1 (secreted by L-cells throughout the intestine) in response to nutrient ingestion act on GIPRs and GLP-1Rs expressed in various organs.*

*GI = gastrointestinal; GIP = gastric inhibitory polypeptide; GIPR = GIP receptor; GLP-1 = glucagon-like peptide; GLP-1R = GLP-1 receptor.*

## GLP-1R activity

GLP-1R, a member of the class B family of G protein-coupled receptors, is found in various organs.^10^ It is expressed mainly in pancreatic β-cells, various gut-cells, and also in the peripheral nervous system and CNS cells (*Figure 1*).^20,21^

Signal transduction occurs after GLP-1R agonists bind to GLP-1Rs, mirroring a GLP-1 bioactivity effect favourable for patients with T2D, where this incretin therapy lowers blood glucose levels by stimulating insulin secretion from β-cells. This therapy is effective when there is no complete loss of β-cell function.^10^ Additionally, there is suppression of appetite induced through the CNS, leading to body weight reduction. Since GLP-1R is expressed in the cells of various organs including pancreatic β-cells, GLP-1R agonists manifest favourable effects during its circulation.^22^

Some low molecular weight GLP-1R agonists can act on GLP-1R in the arcuate nucleus of the hypothalamus, crossing the blood-brain barrier, thereby regulating nerve cells.^23^ In patients prescribed with a GLP-1R agonist as a personalized medicine (that could also reduce the body weight), longer adherence to such therapy with improved satisfaction is anticipated as the patient has chosen a therapy more suitable to their needs.

**Figure 2:**
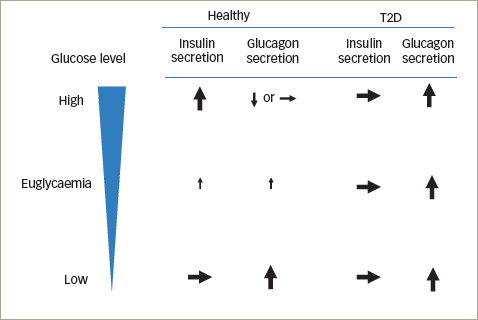
Function of GIP in insulin and glucagon secretion^31–35^

*T2D = type 2 diabetes mellitus.*

## GIP-R activity

GIP is an incretin secreted by the K cells in the upper segment of the small bowel in response to nutrient supply (*Figure 1*).^24^ Following GIP release from the gut endocrine K cells, rapid inactivation occurs by dipeptidyl peptidase-4 (DPP-4) through cleavage into non-insulinotropic truncated forms.^25–27^ The postprandial level of GIP under normal physiological conditions is approximately four times that of GLP-1.^13^ GIP receptors (GIPRs) are present in the β-cells. Additionally, GIPRs are abundantly expressed in adipocytes, the CNS and bones, causing an effect on the adipose tissue, the CNS, and bones (*Figure 1*).^22,28–30^

**Figure 3:**
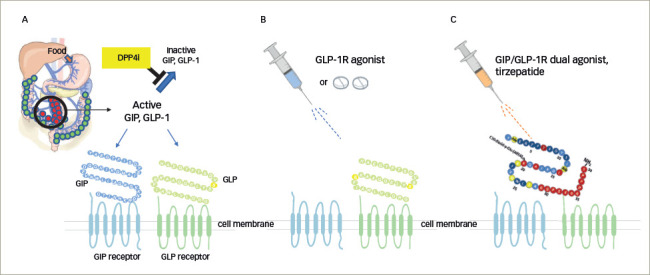
Gut activity in patients with T2D undergoing DPP-4 inhibitor therapy, GLP-1R agonist therapy or GIP/GLP-1R dual agonist therapy^64,66^

*Figure designed by the authors, with reference to Coskun et al. (2018)^66^ and Pelle et al. (2022).^64^*

*A. DPP4i's enhance GIP and GLP-1 which are physiologically derived in the gut in response to oral nutrient intake, thus acting similarly to dual agonists. B. GLP-1R agonists provide mono therapy. C. Tirzepatide, a GIP/GLP-1R dual agonist, relies on the pharmacological levels of GLP-1 and GIP. Residues originated from GIP (blue), GLP-1/exendin-4 (red), and GLP-1 (navy); unique residues are in yellow.*

*C20 diacid-γ-Glu-(AEEA)2 = C20-diacid-γ (gamma) -glutamate-(aminoethylethanolamine)2; DPP4i = dipeptidyl peptidase-4 inhibitor; GIP = gastric inhibitory polypeptide; GLP-1 = glucagon-like peptide-1; GLP-1R = glucagon-like peptide-receptor 1; NH2 = amino group.*

In healthy people, GIP induces nutrient-stimulated insulin and glucagon secretion in a glucose-dependent manner.^14,27^ The stimuli that induce insulin secretion in response to a 50 g oral glucose load are glucose alone (33%), GIP (44%) and GLP-1 (22%).^25,31^ GIP has an augmented effect on insulin secretion, unlike GLP-1, and plays an important role in glucose metabolism in humans.^31^ Moreover, GIP accompanied with insulin or GLP-1 signalling, leads to a reduction in blood glucose levels and body weight. Thus, GIP promotes insulin secretion in healthy individuals, however this effect is not observed in patients with T2D (*Figure 2*).^31–35^

Unlike GLP-1, GIP failed to stimulate glucagon secretion during hyperglycaemia.^33^ Alternatively, stimulated glucagon secretion via GIP does occur during hypoglycaemic conditions accompanied by glucagonotropic enhanced activity.^15,29,30^

The physiological significance of GIP-stimulated glucagon secretion remains unexplored. In contrast, in patients with T2D, glucose-dependency of GIP-stimulated glucagon secretion, such as in healthy people, does not remain (*Figure 2*).^32^ Elimination of GIPR activity in β-cells demonstrated a characteristic of T2D, associated with reduced postprandial insulin secretion and hyperglycaemia (*Figure 2*).

Glucagon secretion by stimulation of GIP, even occurring during hyperglycaemia (*Figure 2*), suggests unfortunate GIPR activity in α-cells, which contributes to the pathogenesis of T2D.^35^ GIP is potentially responsible for the facilitation of glucose-induced β-cell proliferation, in addition to a reduction in α-cell expansion.

GIP positively impacts energy storage by controlling fat metabolism and lipid storage through increased blood flow in adipose tissue and triglyceride uptake; it also accumulates fat via the CNS in humans (*Figure 1*).^36,37^ GIP plays an important role in the regulation of food intake, lipoprotein lipase activity and fat decomposition during carbohydrate/fat degradation in adipose tissue; it produces a direct lipolytic effect during hyperglycaemia and low levels of insulin.^38^

Elimination of GIPR activity by various mechanisms, including genetic ablation blockage, GIPR antagonist or neutralization of GIPR, has a favourable effect on glucose tolerance and obesity.^39–42^ GIPR antagonists have been developed as treatments for T2D and obesity.^43^ Additionally, enhancement of GIP activity, using agonists of GIPR or chronic elevation of GIP levels causing weight loss when coexisting with GLP-1 receptor activation, are useful treating obesity and T2D, leading to improved insulin secretion, glycaemic control, and an accompanying amelioration of β-cell function.^43–47^ Further studies on clinical use of tirzepatide associated with GIPR activity will provide a platform for unravelling the pathogenesis and understanding GIPR activity in α-cells and regulation of glucagon secretion.

GIP-based pharmacotherapy, which relies on CNS–GIPR signalling, is of importance, particularly in systemic metabolic control.^28,29^ Apoptosis inhibition and osteoblast proliferation are also stimulated by GIP, resulting in bone formation and remodelling.^30^

## Protective effect of incretin on various organs

Any long-acting GLP-1R agonist exhibits the same anti-inflammatory effects as natural GLP-1, and is therefore demonstrated to protect organs such as the heart, kidneys and others (*Figure 1*).^48–52^ GLP-1 and other intestinal hormones are known to drive natriuresis via the gut-kidney axis.^53–55^ Onset of apparent albuminuria in high-risk patients with T2D having cerebro-cardiovascular disease complications was significantly reduced by long-acting GLP-1R agonist administration.^49,54,55^ The reduction in cerebrovascular events was significantly noted among Asian patients.^56^ GIP has also been shown to reduce pro-inflammatory cytokine expression, thereby reducing the inflammatory response in the brain. The beneficial effects of GIP on neurodegenerative diseases are well documented.^57^ Pharmacological concentrations of GIP agonists are more likely to provide protective effects against atherosclerosis in both patients with diabetic and non-diabetic conditions.

**Figure 4:**
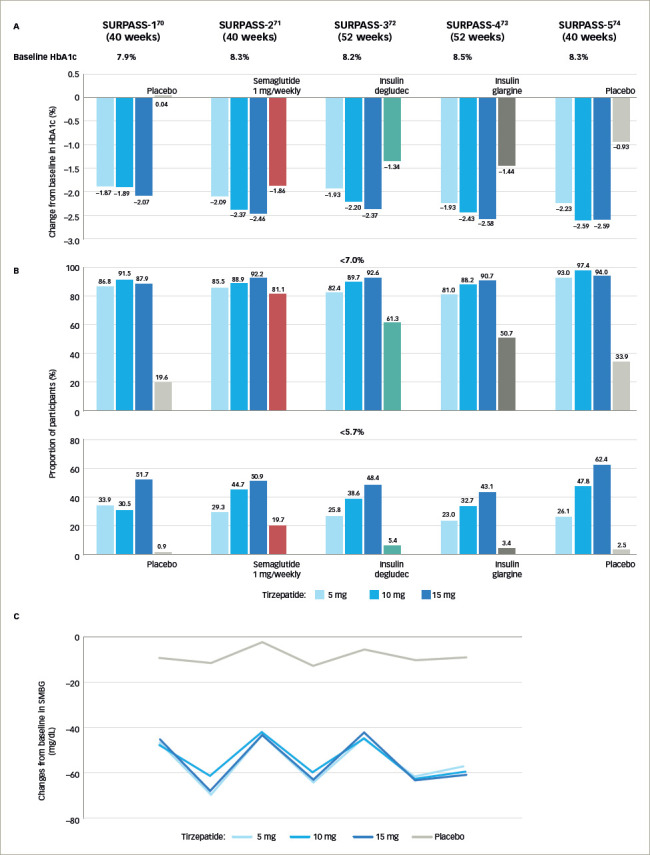
Results from the SURPASS clinical trials^70–74^

*Figure designed by the authors from the clinical trial data from SURPASS 1–5.^70–84^ (A) Change from baseline in HbA1c (%) in SURPASS-1–5. (B) Participants reaching HbA1c target (%) in SURPASS-1-5. (C) Seven-point changes from baseline in SMBG (mg/dL) in SURPASS-1.*

*HbA1c = glycated haemoglobin A1C; SMGB = self-monitoring blood glucose.*

**Table 1:** SURPASS phase III clinical trials for patients with T2D^70–80^

Study acronym	Study type	Number of participants	Eligibility	Comparator	Study duration (weeks)	Primary outcome
SURPASS-1^70^	Randomized double-blind	478	Drug-naïve	Placebo	40	HbA1c
SURPASS-2^71^	Randomized open-label	1,879	Metformin	Semaglutide	40	HbA1c
SURPASS-3^72^	Randomized open-label	1,947	Metformin w/wo sGLT2i	Insulin degludec	52	HbA1c
SURPASS-4^73^	Randomized open-label	2,002	1–3 antidiabetic medicines (metformin, SGLTi or sulfonylurea) with cardiovascular risk	Insulin glargine	52	HbA1c
SURPASS-5^74^	Randomized double-blind	475	Insulin glargine (U100) w/wo metformin	Placebo	40	HbA1c
SURPASS-6^76^	Randomized open-label	1,182	Insulin glargine (U100) w/wo metformin	Insulin lispro	52	HbA1c
SURPASS J-mono^77^	Randomized double-blind	636	Drug-naïve or monotherapy (discontinued before baseline); Japanese	Dulaglutide 0.75 mg	52	HbA1c
SURPASS J-combo^78^	Randomized open-label	443	Non-incretin-based monotherapy; Japanese	N/A	52	Number of participants with SAEs
SURPASS-AP-Combo^79^	Randomized open-label	917	Metformin w/wo sulfonylurea; Asian	Insulin glargine	40	HbA1c
SURPASS-CVOT^80^	Randomized double-blind	12,500	Atherosclerotic cardiovascular disease and overweight	Dulaglutide 1.5 mg	Event driven	Time to MASE-3

*HbA1c = glycated haemoglobin A1C; MASE-3 = death from cardiovascular causes, myocardial Infarction or stroke; SAEs = serious adverse event(s); SGLTI = sodium-glucose cotransporter 2 inhibitor; N/A = not available; w/wo = with or without.*

**Table 2:** Baseline characters and result^21,70–74,76–84^

Study acronym	Age, years (SD)	HbA1c, % (SD)	BMI, kg/m^2^ (SD)	Fasting blood glucose, mg/dL (SD)	eGFR (SD)	Diabetic duration, years (SD)	Other features	Outcome
SURPASS-1^21,70^	54.1 (11.9)	7.9 (0.9) ≤8.5%: 79.0% >8.5%: 21.0%	31.9 (6.6)	154.8 (40.3)	94.1 (19.7)	4.7 (5.4)	White: 35.6%; Native American or Native Alaskan: 24.7%; Asian 35.0%	Superiority
SURPASS-2^71,81^	56.6 (10.4)	8.9 (1.0) ≤8.5%: 63.5% >8.5%: 36.5%	34.2 (6.9)	172.9 (51.46)	96.0 (17.1)	8.6 (6.46)	White 82.6%	Superiority
SURPASS-3^72,82^	57.4 (10.0)	8.2 (0.9)	33.5 (6.1)	169.3 (45.9)	94.1 (17.0)	8.4 (6.2)	White 91.0%	Superiority
SURPASS-4^73,83^	63.6 (8.6)	8.5 (0.9)	32.6 (5.5)	171.2 (50.8)	81.3 (21.1)	10.5 (6.2-15.9)	History of cardiovascular disease 87.0%	Superiority
SURPASS-5^74,84^	60.6 (9.9)	8.3 (0.9)	33.4	162.5	N/A	13.3	White: 80.0%	Superiority
SURPASS-6^76^	Expected read out: August 2022 (information not yet available)							
SURPASS J-mono^77^	Actual study completion date: 31 March 2021 (information not yet available)							
SURPASS J-combo^78^	57.0 (10.8)	8.6 (1.1)	Actual study completion date: 16 February 2021 (information not yet available)					
SURPASS-AP-Combo^79^	Actual study completion date: 24 November 2021 (information not yet available)							
SURPASS-CVOT^80^	Expected read out: 2024 (information not yet available)							

*SD was not provided for all trials, but has been included above where available.*

*BMI = body mass index; eGFR = estimated glomerular filtration rate; HbA1c = glycated haemoglobin A1C; N/A = not available; SD = standard deviation.*

In patients with T2D, the incidence of heart failure significantly increases due to ischaemic heart disease and also separately due to the diabetic condition itself.^58,59^ Obesity, which is closely linked to T2D, is also often associated with heart failure.^60,61^ It is expected that anti-diabetic drugs cause weight reduction, better glycaemic control and improvement in macrovascular outcomes such as cerebral cardiovascular risk factors, in addition to microvascular outcomes.^62,63^

Since evidence suggests that GLP-1R agonists' have a protective effect on various organs (*Figure 1*), the use of a GIP/GLP-1 dual agonist is under clinical investigation to prevent the aggravation of diabetic complications.^50,54,55^

## GIP and GLP-1 synergistic activation

In patients with T2D, GIP-stimulated glucagon secretion observed in healthy participants is not maintained.^34,64^ However, the coexistence of GIP and GLP-1 in patients with T2D produces glucagon concentrations akin to control conditions.^65^ DPP-4 inhibitors enhance GIP and GLP-1, which are physiologically derived in the gut in response to oral nutrient intake, thus acting similarly to the coexistence of GIP and GLP-1 (*Figure 3*).^64,66^

In patients with T2D, physiological secretion of GIP or GLP-1 decreases, thus reducing insulin secretion, compared to that in healthy participants.^28^ Notably, DPP-4 inhibitors enhance GLP-1 or GIP level in patients with T2D. However, in patients with T2D, GIP/GLP-1R dual agonists provide pharmacological concentration levels of GLP-1 and GIP, adding a synergistic effect and producing robust stimulation of insulin secretion.

Pharmacological activation of GIPRs is anticipated to produce therapeutic effects regarding energy metabolism in peripheral tissues, whereas the chronic use of long-acting GIPR agonists did not reduce body weight.^44–46^ Synergistic activation of GIP/GLP-1R produced higher therapeutic efficacy for T2D, leading to a greater body weight reduction, compared with GIP or GLP-1 alone.^43^ Reinforced suppression of calorie intake, accompanied by a gradual increase in energy consumption, produces a reduction in body weight. Evidence suggests that GIP/GLP-1 synergism occurs in the CNS and reduces body weight, independent of insulin sensitivity and fat metabolism.^29^ Compared to a GLP-1R agonist alone, a GIPR/GLP-1R unimolecular dual agonist has also shown restorative and neuroprotective properties.^67^

Regarding the effects of increased antidiabetic action and decreased body weight by dual stimulation of GIP and GLP-1R, more than either GIP or GLP-1 alone, one of the reasons that might be postulated for this synergism is that GIP signals mediate insulinotropic and glucagonotropic effects through the brain or fat. Nevertheless, further research is needed concerning the links between the gut, brain and blood glucose levels which employ gastrointestinal hormones including dual, tri or more synergistic stimulations. Therefore, tirzepatide has the potential to be a key that unlocks the door to this conundrum. While many people have suggested that *in vitro* research has such potential, in fact the direct administration of tirzepatide in humans lends itself to a more timely revealing of the facts behind the system of blood glucose maintenance.^21,31,43,68–74^

## GIP/GLP-1R unimolecular dual agonist, tirzepatide

The GIP/GLP-1R unimolecular dual agonist, tirzepatide, is a multifunctional peptide based on the native GIP peptide sequence, designed to bind both the receptors. The synergistic effect of GIP and GLP-1 on food intake and increasing energy expenditure results in body weight reduction.^20,39^ Tirzepatide structurally comprises a peptide of 39 amino acids (molecular weight: 4810.52 Dalton) with the bioactive sequence of GIP, and with a sequence acting on GLP-1 replacing its intermediate amino acid.^1–4,20,68^ In comparison to native GLP-1 with one-fifth affinity, tirzepatide has an affinity similar to that of native GIP.^20^ Tirzepatide's once-weekly administration derives from its structure, which is based on the GIP sequence and includes a C20 fatty di-acid moiety, enabling it to bind to albumen and prolong its half-life of 5 days.^68^

Tirzepatide has exhibited robust results in glycaemic control and body weight reduction compared to other alternatives.^68^ In my opinion, early stage T2D results from the ‘misalignment of the metabolic orbits' of insulin, glucagon, intestinal hormones such as representative incretins, and other factors in the CNS.^68^ Since conventional treatment of T2D is not aimed at restoring original pancreatic β-cell function, but instead ‘exogenously replenishing’ the declining function, tirzepatide robust effectiveness is due to the improvement of the misalignment of the metabolic orbits as it might act as a critical factor of metabolic orbit. This improvement in misalignment may be also possibly due to intervention in GIP signalling with GLP-1 signalling.

## SURPASS clinical trials

**Figure 5:**
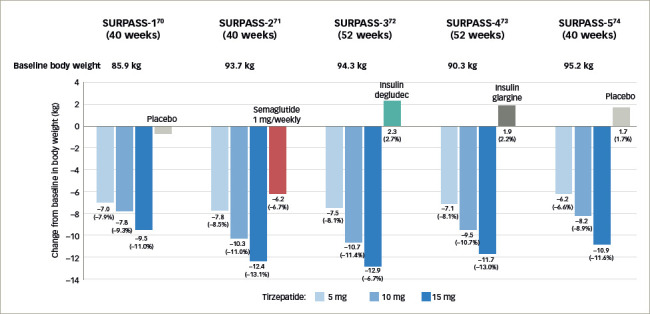
Change from baseline in body weight in SURPPASS21,^70–74,81–84^

In the original phase II trial (A phase 2 study of once-weekly LY3298176 compared with placebo and dulaglutide in patients with type 2 diabetes mellitus; ClinicalTrials.gov identifier: NCT03131687), patients with T2D with diet and exercise therapy alone or those undergoing metformin therapy, and a BMI of 23–50 kg/m^2^, were randomly assigned to receive either once-weekly tiazepatide (1 mg, 5 mg, 10 mg or 15 mg), dulaglutide (1.5 mg), or placebo for 26 weeks.^69,75^ Based on the primary efficacy outcome from this study - that is, change in glycated haemoglobin A1C (HbA1c) from baseline to 26 weeks - the SURPASS clinical phase III trials (A phase of tirzepatide [LY3298176] in participants with type 2 diabetes not controlled with diet and exercise alone (SURPASS-1); ClinicalTrials. gov identifier: NCT03954834; A study of tirzepatide [LY3298176] versus semaglutide once weekly as add-on therapy to metformin in participants with type 2 diabetes [SURPASS-2]; ClinicalTrials.gov identifier: NCT03987919; A study of tirzepatide (LY3298176) versus insulin degludec in participants with type 2 diabetes [SURPASS-3]; ClinicalTrials. gov identifier: NCT03882970; A study of tirzepatide (LY3298176) once a week versus insulin glargine once a day in participants with type 2 diabetes and increased cardiovascular risk [SURPASS-4] ClinicalTrials.gov identifier: NCT03730662) employed 3 dosages.^69–73^ Global phase III clinical trials conducted to evaluate the efficacy and safety of weekly tirzepatide included anti-hyperglycaemic therapy-naive patients and patients on various oral anti-hyperglycaemic drugs, including metformin, sulfonylurea, pioglitazone, SGLT2 inhibitor and/or insulin compared with placebo, basal insulins, or the conventional GLP-1R agonist (*Tables 1* and *2*, *Figure 4*).^21,70–74,76–84^ Tirzepatide demonstrated robust glycaemic control (*Figure 4*) and body weight reduction compared to the controls, which included the conventional GLP-1R agonist semaglutide. In particular, in the SURPASS-1 trial, tirzepatide was reported to be a curative drug in patients with early stage T2D. In all studies, over 90% of the patients taking tirzepatide achieved HbA1c levels <7%; between 40% and 52% of patients achieved HbA1c levels <5.7% (*Figure 4*).^70–74^ Weight reduction ranged from 6.2–12.9 kg (6.6–13.9%) depending on the dose (*Figure 5*); in addition, this weight reduction did not bottom out at 40 weeks.^21,70–74,81–84^ Weight loss of 15% or more was observed in 13–27% of patients.^70–74^

*Graphs above constructed from the clinical trial data from SURPASS 1–5.^21,70–74,81–84^*

**Table 3:** Other clinical trials with tirzepatide^86–88^

Study acronym	Study type	Number of enrolments	Eligibility	Comparator	Comparator Study duration (weeks)	Primary outcome	Estimated completion
SUMMIT (NCT04847557)^86^	Phase III randomized double-blind	Estimated 700	≥40 years old, stable heart failure (NYHA class II-IV) and LVEF ≥50%	Placebo	52	Efficacy and safety	November 2023
SURMOUNT-1 (NCT04184622)^87^	Phase III randomized double-blind	2,539	BMI ≥30 kg/m^2^, or BMI ≥27 kg/m^2^ with related comorbidities	Placebo	72	Weight decrease in people with diabetes and obesity	May 2024
SYNERGY-NASH (NCT04166773)^88^	Phase II randomized double-blind	Estimated 196	BMI ≥27 kg/m^2^ and NASH stage 2 or 3 fibrosis	Placebo	52	Absence of NASH, with no worsening of fibrosis on liver histology	December 2023

*BMI = body mass index; LVEF = left ventricular ejection fraction; NASH = non-alcoholic steatohepatitis; NYHA = New York Heart Association.*

It has been reported that post-bariatric surgery decreases in body weight were -45.0 kg at 2 years, -36.3 kg at 6 years and -35.0 kg at 12 years, while the decreases in those not undergoing the surgery were -2.9 kg at 2 years and 0 kg at 12 years. However, surgery for severe obesity generally brings a high level of risk. Firstly, two-step treatment, such as GIP/GLP-1R agonist treatment following bariatric surgery would provide a more effective outcome than each therapy undertaken alone. Tirzepatide treatment has also been studied in clinical trials for cardiovascular (CV) outcomes (*Tables 1* and *2*). The full list of SURPASS trials is as follows:

- SURPASS-1 (ClinicalTrials.gov identifier: NCT03954834): assesses efficacy, safety, and tolerability of tirzepatide monotherapy versus placebo in patients with T2D with diet and exercise alone.^70^
- SURPASS-2 (ClinicalTrials.gov identifier: NCT03987919): assesses efficacy and safety of tirzepatide versus semaglutide (1 mg) weekly as add-on therapy to metformin in patients with T2D.^71^
- SURPASS-3 (ClinicalTrials.gov identifier: NCT03882970): assesses efficacy and safety of tirzepatide versus insulin degludec in patients with T2D prescribed metformin with or without SGLT2 inhibitors.^72^
- SURPASS-4 (ClinicalTrials.gov identifier: NCT03730662): assesses the efficacy and safety of tirzepatide versus insulin glargine in patients with T2D with high CV risk (87% of participants had previous events) on oral anti-hyperglycaemic medications.^73^
- SURPASS-5 (A study of tirzepatide (LY3298176) versus placebo in participants with type 2 diabetes inadequately controlled on insulin glargine with or without metformin; ClinicalTrials.gov identifier: NCT04039503): assesses the efficacy and safety of the addition of tirzepatide versus placebo in patients with T2D prescribed insulin glargine with or without metformin.^74^
- SURPASS-6 (A study of tirzepatide (LY3298176) versus insulin lispro (U100) in participants with type 2 diabetes inadequately controlled on insulin glargine (U100) with or without metformin; ClinicalTrials.gov identifier: NCT04537923): assesses the effect of the addition of tirzepatide weekly versus insulin lispro (U100) three times daily in patients with T2D prescribed insulin glargine (U100) with or without metformin. This study has an anticipated read-out in 2022.^76^
- SURPASS J-mono (A study of tirzepatide (LY3298176) compared to dulaglutide in participants with type 2 diabetes; ClinicalTrials.gov identifier: NCT03861052): assesses the efficacy and safety of tirzepatide monotherapy versus dulaglutide (0.75 mg) weekly in patients with T2D for drug approval in Japan.^77^
- SURPASS J-combo (A long-term safety study of tirzepatide (LY3298176) in participants with type 2 diabetes; ClinicalTrials.gov identifier: NCT03861039): assesses the long-term safety of tirzepatide in combination with monotherapy of oral antihyperglycemic medications in patients with T2D for drug approval in Japan.^78^
- SURPASS-AP-Combo (A study of tirzepatide (LY3298176) in participants with type 2 diabetes on metformin with or without sulfonylurea; ClinicalTrials.gov identifier: NCT04093752): assesses the efficacy of tirzepatide versus insulin glargine in Asian patients with T2D prescribed metformin with or without a sulfonylurea.^79^

**Figure 6:**
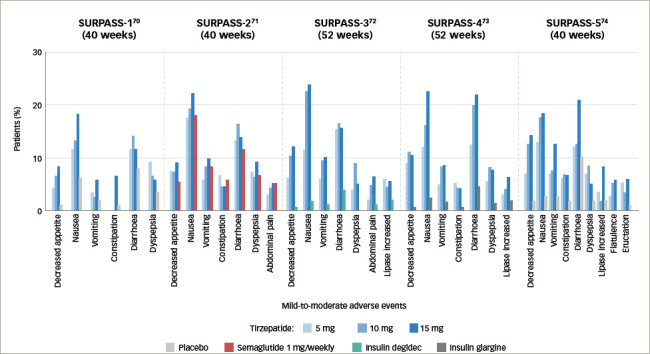
Mild-to-moderate adverse events in SURPASS^70–74^

*Graphs above constructed from the clinical trial data from SURPASS 1–5.^70–74^*

## Clinical trials for cardiovascular outcomes

Regulatory submission requirements for the evaluation of the cardiovascular risk hazard ratio of 0.81 collected from pooled SURPASS data regarding four-component major adverse cardiac event (MACE-4) events (cardiovascular death, myocardial infarction, stroke and hospitalized unstable angina) were met by the SURPASS program.^85^ Most MACE-4 events that were recognised were seen in SURPASS-4, suggesting a hazard ratio of 0.74 (95% CI, 0.51–1.08) (p=0.123).^83^

The global SURPASS-CVOT (A study of tirzepatide (LY3298176) compared with dulaglutide on major cardiovascular events in participants with type 2 diabetes; ClinicalTrials.gov Identifier: NCT04255433) assesses the noninferiority and superiority of tirzepatide against dulaglutide (1.5 mg dose weekly) with a confirmed cardio-protective effect.^80^ Patients with T2D and increased CV risk were enrolled.^80^ This trial was started in June 2020, with an expected read-out in 2024. The phase III SUMMIT study (A study of tirzepatide (LY3298176) in participants with heart failure with preserved ejection fraction and obesity; ClinicalTrials.gov identifier: NCT04847557) was initiated in 2021 (*Table 3*).^86–88^ The main purpose of this study is to assess the efficacy and safety of tirzepatide in participants suffering from heart failure with preserved ejection fraction and obesity.

## Other tirzepatide trials

The other trials into tirzepatide are as follows (*Table 3*):

- SUMMIT (ClinicalTrials.gov Identifier: NCT04847557): a phase III trial comparing the efficacy and safety of tirzepatide versus placebo in patients with heart failure with preserved ejection fraction, as described above.^86^
- SURMOUNT-1 (A study of tirzepatide (LY3298176) in participants with obesity or overweight; ClinicalTrials.gov Identifier: NCT04184622): a phase III trial evaluating the efficacy of tirzepatide in reducing weight in obese subjects without T2D at 72 weeks. Eligible participants were those with either a BMI ≥30 kg/m^2^, or ≥27 kg/m^2^ plus one or more of the following: hypertension, dyslipidemia, obstructive sleep apnea or cardiovascular disease.^87^
- SYNERGY-NASH (ClinicalTrials.gov Identifier: NCT04166773): a phase II trial for the efficacy and safety of tirzepatide in participants with non-alcoholic steatohepatitis (NASH). The primary outcome is the proportion of the participants with an absence of NASH at 52 weeks with no worsening of fibrosis in liver pathology. Eligible participants were those with a BMI ≥27 kg/ m^2^ and ≤50 kg/m^2^, with or without T2D and having a histologic diagnosis of NASH with stage 2 or 3 fibrosis following liver biopsy.^88^

## Safety profile

There are no specific signs in a serious adverse event. Mild-to-moderate adverse effects (including nausea, vomiting and diarrhoea) occurred frequently during the dose-escalation period across all doses of tirzepatide in the SURPASS studies (*Figure 6*).^70–74^ Mild-to-moderate gastrointestinal adverse effects (including nausea, appetite loss, diarrhoea and constipation) were similar to those observed with conventional incretin preparations (including semaglutide), but were more frequent with tirzepatide than with semaglutide.^70–74^ This was transient, as the trial drug was discontinued in 3–11% of the patients. When tirzepatide was not combined with sulfonylurea or insulin, the occurrence of hypoglycaemia remained low.^70–74^

## Conclusion

Tirzepatide, a dual GIP/GLP-1R agonist, demonstrated robust reductions in glycaemic control and body weight for the patients in the SURPASS studies, without causing hypoglycaemia. The current focus areas for research are preventative factors to combat accelerated β-cell deterioration - a critical cause of diabetes - and exploring the benefits of GIPR. The novel drugs represented by tirzepatide seem promising in revealing new pathogenic insights. It is also believed that unexploited anti-diabetic drugs with GIP and GLP-1 signal activation will unravel undiscovered avenues of treatment for T2D and its complications. The clinical success of this dual GIP/GLP-1R agonist will inspire the development of multiple agonists, such as other dual agonists or triagonists consisting of multiple ligands, including hormones with wider applications.

## References

[1] Nauck M, Stockmann F, Ebert R, Creutzfeldt W (1986). Reduced incretin effect in type 2 (non-insulin-dependent) diabetes.. Diabetologia..

[2] Krarup T, Saurbrey N, Moody AJ (1987). Effect of porcine gastric inhibitory polypeptide on beta-cell function in type I and type II diabetes mellitus.. Metabolism..

[3] Takeda J, Seino Y, Tanaka K (1987). Sequence of an intestinal cDNA encoding human gastric inhibitory polypeptide precursor.. Proc Natl Acad Sci USA..

[4] Inagaki N, Seino Y, Takeda J (1989). Gastric inhibitory polypeptide: Structure and chromosomal localization of the human gene.. Mol Endocrinol..

[5] Miyawaki K, Yamada Y, Ban N (2002). Inhibition of gastric inhibitory polypeptide signalling prevents obesity.. Nat Med..

[6] Harada N, Hamasaki A, Yamane S (2011). Plasma gastric inhibitory polypeptide and glucagon-like peptide-1 levels after glucose loading are associated with different factors in Japanese subjects.. J Diabetes Investig..

[7] Nauck MA, Homberger E, Siegel EG (1986). Incretin effects of increasing glucose loads in man calculated from venous insulin and C-peptide responses.. J Clin Endocrinol Metab..

[8] Andersen A, Lund A, Knop FK, Vilsboll T (2018). Glucagon-like peptide 1 in health and disease.. Nat Rev Endocrinol..

[9] Fujita Y, Asadi A, Yang GK (2010). Differential processing of pro-glucose-dependent insulinotropic polypeptide in gut.. Am J Physiol Gastrointest Liver Physiol..

[10] Campbell JE, Drucker DJ (2013). Pharmacology, physiology, and mechanisms of incretin hormone action.. Cell Metab..

[11] Richards P, Parker HE, Adriaenssens AE (2014). Identification and characterization of GLP-1 receptor-expressing cells using a new transgenic mouse model.. Diabetes..

[12] Gabery S, Salinas CG, Paulsen SJ (2020). Semaglutide lowers body weight in rodents via distributed neural pathways.. JCI Insight..

[13] Vollmer K, Holst JJ, Baller B (2008). Predictors of incretin concentrations in subjects with normal, impaired, and diabetic glucose tolerance.. Diabetes..

[14] Adriaenssens AE, Biggs EK, Darwish T (2019). Glucose-dependent insulinotropic polypeptide receptor-expressing cells in the hypothalamus regulate food Intake.. Cell Metab..

[15] Christensen M, Vedtofte L, Holst JJ (2011). Glucose-dependent insulinotropic polypeptide: A bifunctional glucose-dependent regulator of glucagon and insulin secretion in humans.. Diabetes..

[16] Dunning BE, Gerich JE (2007). The role of alpha-cell dysregulation in fasting and postprandial hyperglycemia in type 2 diabetes and therapeutic implications.. Endocr Rev..

[17] Campbell JE, Drucker DJ (2015). Islet alpha cells and glucagon--critical regulators of energy homeostasis.. Nat Rev Endocrinol..

[18] Brandt SJ, Gotz A, Tschop MH, Muller TD (2018). Gut hormone polyagonists for the treatment of type 2 diabetes.. Peptides..

[19] Kalra S, Sahay R (2020). A review on semaglutide: An oral glucagon-like peptide 1 receptor agonist in management of type 2 diabetes mellitus.. Diabetes Ther..

[20] Tschop MH, Finan B, Clemmensen C (2016). Unimolecular polypharmacy for treatment of diabetes and obesity.. Cell Metab..

[21] Rosenstock J, Wysham C, Frías JP (2021). Efficacy and safety of a novel dual GIP and GLP-1 receptor agonist tirzepatide in patients with type 2 diabetes (SURPASS-1): A double-blind, randomised, phase 3 trial.. Lancet..

[22] Pyke C, Heller RS, Kirk RK (2014). GLP-1 receptor localization in monkey and human tissue: Novel distribution revealed with extensively validated monoclonal antibody.. Endocrinology..

[23] Nauck MA, Meier JJ (2018). Incretin hormones: Their role in health and disease.. Diabetes Obes Metab..

[24] Usdin TB, Mezey E, Button DC (1993). Gastric inhibitory polypeptide receptor, a member of the secretin-vasoactive intestinal peptide receptor family, is widely distributed in peripheral organs and the brain.. Endocrinology..

[25] Dupre J, Ross SA, Watson D, Brown JC (1973). Stimulation of insulin secretion by gastric inhibitory polypeptide in man.. J Clin Endocrinol Metab..

[26] Schirra J, Katschinski M, Weidmann C (1996). Gastric emptying and release of incretin hormones after glucose ingestion in humans.. J Clin Invest..

[27] Pauly RP, Rosche F, Wermann M (1996). Investigation of glucose-dependent insulinotropic polypeptide-(1-42) and glucagon-like peptide-1-(7-36) degradation in vitro by dipeptidyl peptidase IV using matrix-assisted laser desorption/ionization-time of flight mass spectrometry. A novel kinetic approach.. J Biol Chem..

[28] Deacon CF, Nauck MA, Meier J (2000). Degradation of endogenous and exogenous gastric inhibitory polypeptide in healthy and in type 2 diabetic subjects as revealed using a new assay for the intact peptide.. J Clin Endocrinol Metab..

[29] Zhang Q, Delessa CT, Augustin R (2021). The glucose-dependent insulinotropic polypeptide (GIP) regulates body weight and food intake via CNS-GIPR signaling.. Cell Metab..

[30] Bollag RJ, Zhong Q, Phillips P (2000). Osteoblast-derived cells express functional glucose-dependent insulinotropic peptide receptors.. Endocrinology..

[31] Nauck MA, Meier JJ (2019). GIP and GLP-1: Step siblings rather than monozygotic twins within the incretin family.. Diabetes..

[32] Nauck MA, Heimesaat MM, Orskov C (1993). Preserved incretin activity of glucagon-like peptide 1 [7-36 amide] but not of synthetic human gastric inhibitory polypeptide in patients with type-2 diabetes mellitus.. J Clin Invest..

[33] El K, Campbell JE (2020). The role of GIP in alpha-cells and glucagon secretion.. Peptides..

[34] Meier JJ, Gallwitz B, Siepmann N (2003). Gastric inhibitory polypeptide (GIP) dose-dependently stimulates glucagon secretion in healthy human subjects at euglycaemia.. Diabetologia..

[35] Christensen MB, Calanna S, Holst JJ (2014). Glucose-dependent insulinotropic polypeptide: Blood glucose stabilizing effects in patients with type 2 diabetes.. J Clin Endocrinol Metab..

[36] Asmar M, Asmar A, Simonsen L (2017). The gluco- and liporegulatory and vasodilatory effects of glucose-dependent insulinotropic polypeptide (GIP) are abolished by an antagonist of the human GIP receptor.. Diabetes..

[37] Samms RJ, Sloop KW, Gribble FM (2021). GIPR function in the central nervous system: Implications and novel perspectives for GIP-based therapies in treating metabolic disorders.. Diabetes..

[38] Heimburger SMN, Nielsen CN, Calanna S (2022). Glucose-dependent insulinotropic polypeptide induces lipolysis during stable basal insulin substitution and hyperglycaemia in men with type 1 diabetes: A randomized, double-blind, placebo-controlled, crossover clinical trial.. Diabetes Obes Metab..

[39] Killion EA, Wang J, Yie J (2018). Anti-obesity effects of GIPR antagonists alone and in combination with GLP-1R agonists in preclinical models.. Sci Transl Med..

[40] Gault VA, Irwin N, Green BD (2005). Chemical ablation of gastric inhibitory polypeptide receptor action by daily (Pro3)GIP administration improves glucose tolerance and ameliorates insulin resistance and abnormalities of islet structure in obesity-related diabetes.. Diabetes..

[41] Svendsen B, Capozzi ME, Nui J (2020). Pharmacological antagonism of the incretin system protects against diet-induced obesity.. Mol Metab..

[42] Gault VA, O’Harte FP, Harriott P (2003). Effects of the novel (Pro3)GIP antagonist and exendin(9-39)amide on GIP- and GLP-1-induced cyclic AMP generation, insulin secretion and postprandial insulin release in obese diabetic (ob/ob) mice: evidence that GIP is the major physiological incretin.. Diabetologia..

[43] Holst JJ, Rosenkilde MM (2020). GIP as a therapeutic target in diabetes and obesity: Insight from incretin co-agonists.. J Clin Endocrinol Metab..

[44] Mroz PA, Finan B, Gelfanov V (2019). Optimized GIP analogs promote body weight lowering in mice through GIPR agonism not antagonism.. Mol Metab..

[45] Norregaard PK, Deryabina MA, Tofteng Shelton P (2018). A novel GIP analogue, ZP4165, enhances glucagon-like peptide-1-induced body weight loss and improves glycaemic control in rodents.. Diabetes Obes Metab..

[46] Irwin N, Green BD, Mooney MH (2005). A novel, long-acting agonist of glucose-dependent insulinotropic polypeptide suitable for once-daily administration in type 2 diabetes.. J Pharmacol Exp Ther..

[47] Widenmaier SB, Kim SJ, Yang GK (2010). A GIP receptor agonist exhibits beta-cell anti-apoptotic actions in rat models of diabetes resulting in improved beta-cell function and glycemic control.. PLoS One..

[48] Gerstein HC, Colhoun HM, Dagenais GR (2019). Dulaglutide and cardiovascular outcomes in type 2 diabetes (REWIND): A double-blind, randomised placebo-controlled trial.. Lancet..

[49] Marso SP, Bain SC, Consoli A (2016). Semaglutide and cardiovascular outcomes in patients with type 2 diabetes.. N Engl J Med..

[50] Fujita H, Morii T, Fujishima H (2014). The protective roles of GLP-1R signaling in diabetic nephropathy: Possible mechanism and therapeutic potential.. Kidney Int..

[51] Muskiet MHA, Tonneijck L, Smits MM (2017). GLP-1 and the kidney: From physiology to pharmacology and outcomes in diabetes.. Nat Rev Nephrol..

[52] Rizzo M, Abate N, Chandalia M (2015). Liraglutide reduces oxidative stress and restores heme oxygenase-1 and ghrelin levels in patients with type 2 diabetes: A prospective pilot study.. J Clin Endocrinol Metab..

[53] Zhang H, Zhang X, Hu C, Lu W (2012). Exenatide reduces urinary transforming growth factor-beta1 and type IV collagen excretion in patients with type 2 diabetes and microalbuminuria.. Kidney Blood Press Res..

[54] Husain M, Bain SC, Jeppesen OK (2020). Semaglutide (SUSTAIN and PIONEER) reduces cardiovascular events in type 2 diabetes across varying cardiovascular risk.. Diabetes Obes Metab..

[55] Marso SP, Daniels GH, Brown-Frandsen K (2016). Liraglutide and cardiovascular outcomes in type 2 diabetes.. N Engl J Med..

[56] Lee MMY, Ghouri N, McGuire DK (2021). Meta-analyses of results from randomized outcome trials comparing cardiovascular effects of SGLT2is and GLP-1RAs in Asian versus white patients with and without type 2 diabetes.. Diabetes Care..

[57] Zhang ZQ, Holscher C (2020). GIP has neuroprotective effects in Alzheimer and Parkinson’s disease models.. Peptides..

[58] Kannel WB, Hjortland M, Castelli WP (1974). Role of diabetes in congestive heart failure: The Framingham study.. Am J Cardiol..

[59] Nichols GA, Hillier TA, Erbey JR, Brown JB (2001). Congestive heart failure in type 2 diabetes: Prevalence, incidence, and risk factors.. Diabetes Care..

[60] Brookheart RT, Michel CI, Schaffer JE (2009). As a matter of fat.. Cell Metab..

[61] Shah AD, Langenberg C, Rapsomaniki E (2015). Type 2 diabetes and incidence of cardiovascular diseases: A cohort study in 1.9 million people.. Lancet Diabetes Endocrinol..

[62] Mentis N, Vardarli I, Kothe LD (2011). GIP does not potentiate the antidiabetic effects of GLP-1 in hyperglycemic patients with type 2 diabetes.. Diabetes..

[63] Seidu S, Cos X, Brunton S (2022). 2022 update to the position statement by Primary Care Diabetes Europe: A disease state approach to the pharmacological management of type 2 diabetes in primary care.. Prim Care Diabetes..

[64] Pelle CM PM, Zaffina I, Pujia R (2022). Role of a dual glucose-dependent insulinotropic peptide (GIP)/glucagon-like peptide-1 receptor agonist (twincretin) in glycemic control: From pathophysiology to treatment.. Life (Basel)..

[65] Park H, Park C, Kim Y, Rascati KL (2012). Efficacy and safety of dipeptidyl peptidase-4 inhibitors in type 2 diabetes: Meta-analysis.. Ann Pharmacother..

[66] Coskun T, Sloop KW, Loghin C (2018). LY3298176, a novel dual GIP and GLP-1 receptor agonist for the treatment of type 2 diabetes mellitus: From discovery to clinical proof of concept.. Mol Metab..

[67] Salles GN, Calio ML, Holscher C (2020). Neuroprotective and restorative properties of the GLP-1/GIP dual agonist DA-JC1 compared with a GLP-1 single agonist in Alzheimer’s disease.. Neuropharmacology..

[68] Kaneko S (2021). Novel approaches to pharmacological management of type 2 diabetes in Japan.. Expert Opin Pharmacother..

[69] Frias JP, Nauck MA, Van J (2018). Efficacy and safety of LY3298176, a novel dual GIP and GLP-1 receptor agonist, in patients with type 2 diabetes: A randomised, placebo-controlled and active comparator-controlled phase 2 trial.. Lancet..

[70] ClinicalTrials.gov. A Study of Tirzepatide (LY3298176) in Participants With Type 2 Diabetes Not Controlled With Diet and Exercise Alone (SURPASS-1). ClinicalTrials.gov Identifier: NCT03954834.. NCT03954834.

[71] ClinicalTrials.gov. A Study of Tirzepatide (LY3298176) Versus Semaglutide Once Weekly as Add-on Therapy to Metformin in Participants With Type 2 Diabetes (SURPASS-2). ClinicalTrials. gov Identifier: NCT03987919.. NCT03987919.

[72] ClinicalTrials.gov. A Study of Tirzepatide (LY3298176) Versus Insulin Degludec in Participants With Type 2 Diabetes (SURPASS-3). ClinicalTrials.gov Identifier: NCT03882970.. NCT03882970.

[73] ClinicalTrials.gov. Efficacy and Safety of LY3298176 Once Weekly Versus Insulin Glargine in Patients With Type 2 Diabetes and Increased Cardiovascular Risk (SURPASS-4). ClinicalTrials. gov Identifier: NCT03730662.. NCT03730662.

[74] ClinicalTrials.gov. A Study of Tirzepatide (LY3298176) Versus Placebo in Participants With Type 2 Diabetes Inadequately Controlled on Insulin Glargine With or Without Metformin (SURPASS-5). ClinicalTrials.gov Identifier: NCT04039503.. NCT04039503.

[75] ClinicalTrials.gov. A Study of Tirzepatide (LY3298176) in Participants With Type 2 Diabetes Mellitus. ClinicalTrials.gov Identifier: NCT03131687.. NCT03131687.

[76] ClinicalTrials.gov. A Study of Tirzepatide (LY3298176) Versus Insulin Lispro (U100) in Participants With Type 2 Diabetes Inadequately Controlled onInsulin Glargine (U100) With or Without Metformin (SURPASS-6). ClinicalTrials.gov Identifier: NCT04537923.. NCT04537923.

[77] ClinicalTrials.gov. A Study of Tirzepatide (LY3298176) Compared to Dulaglutide in Participants With Type 2 Diabetes (SURPASS J-mono). ClinicalTrials.gov Identifier: NCT03861052.. NCT03861052.

[78] ClinicalTrials.gov. A Long-term Safety Study of Tirzepatide (LY3298176) in Participants With Type 2 Diabetes. ClinicalTrials.gov Identifier: NCT03861039.. NCT03861039.

[79] ClinicalTrials.gov. A Study of Tirzepatide (LY3298176) in Participants With Type 2 Diabetes on Metformin With or Without Sulfonylurea (SURPASS-AP-Combo). ClinicalTrials.gov Identifier: NCT04093752.. NCT04093752.

[80] ClinicalTrials.gov. A Study of Tirzepatide (LY3298176) Compared With Dulaglutide on Major Cardiovascular Events in Participants With Type 2 Diabetes (SURPASS-CVOT). ClinicalTrials.gov Identifier: NCT04255433.. NCT04255433.

[81] Frias JP, Davies MJ, Rosenstock J (2021). Tirzepatide versus semaglutide once weekly in patients with type 2 diabetes.. N Engl J Med..

[82] Ludvik B, Giorgino F, Jodar E (2021). Once-weekly tirzepatide versus once-daily insulin degludec as add-on to metformin with or without SGLT2 inhibitors in patients with type 2 diabetes (SURPASS-3): a randomised, open-label, parallel-group, phase 3 trial.. Lancet..

[83] Del Prato S, Kahn SE, Pavo I (2021). Tirzepatide versus insulin glargine in type 2 diabetes and increased cardiovascular risk (SURPASS-4): A randomised, open-label, parallel-group, multicentre, phase 3 trial.. Lancet..

[84] Dahl D, Onishi Y, Norwood P (2021). Tirzepatide, a dual GIP/GLP-1 receptor agonist, is effective and safe when added to basal insulin for treatment of type 2 diabetes (SURPASS-5).. Diabetologia..

[85] Sattar N, McGuire DK, Pavo I (2022). Tirzepatide cardiovascular event risk assessment: A pre-specified meta-analysis.. Nat Med..

[86] ClinicalTrials.gov. A Study of Tirzepatide (LY3298176) in Participants With Heart Failure With Preserved Ejection Fraction and Obesity (SUMMIT) (SUMMIT). ClinicalTrials.gov Identifier: NCT04847557.. NCT04847557.

[87] ClinicalTrials.gov. A Study of Tirzepatide (LY3298176) in Participants With Obesity or Overweight (SURMOUNT-1). ClinicalTrials.gov Identifier: NCT04184622.. NCT04184622.

[88] ClinicalTrials.gov. A Study of Tirzepatide (LY3298176) in Participants With Nonalcoholic Steatohepatitis (NASH) (SYNERGY-NASH). ClinicalTrials.gov Identifier: NCT04166773.. NCT04166773.

